# A versatile method for assessing pathogenicity of *Hymenoscyphus fraxineus* to ash foliage

**DOI:** 10.1111/efp.12484

**Published:** 2018-12-14

**Authors:** Elizabeth S. Orton, Martha Clarke, Clive M. Brasier, Joan F. Webber, James K. M. Brown

**Affiliations:** ^1^ John Innes Centre Norwich Research Park Norwich UK; ^2^ Forest Research Alice Holt Lodge Farnham UK

## Abstract

We describe a method for inoculating rachises of *Fraxinus excelsior* (European or common ash) with *Hymenoscyphus fraxineus,* which is faster than previous methods and allows associated foliar symptoms to be assessed on replicate leaves. A total of ten ash seedlings were inoculated with five isolates of *H. fraxineus* and lesion development assessed over four weeks. A five‐point disease progress scale of symptom development was developed from no lesion (0), lesion on rachis (1), “pre‐top dead,” with curling of distal leaflets and bending of the rachis (2), top dead, with wilting and death of distal leaflets (3) to leaf abscission (4). The method revealed variation in aggressiveness of *H. fraxinus* isolates and may be suitable for assessing the resistance of *F. excelsior* and other *Fraxinus* species to dieback. The in vitro growth rate of *H. fraxineus* isolates was highly correlated with both disease progress and the length of rachis lesions on susceptible plants, indicating that it can be used as a preliminary step in selecting isolates with high aggressiveness for use in resistance screening.

## 1 INTRODUCTION

Ash dieback disease, caused by *Hymenoscyphus fraxineus* has devastated common (European) ash trees (*Fraxinus excelsior*) across Europe. The fungus is native to eastern Asia and appears to have little pathogenicity to the indigenous ash species *F. mandshurica* in its native range, whereas it is extremely pathogenic on most *F. excelsior* trees (Gross, Zaffarano, Duo, & Grunig, 2012). The pathogen is wind‐dispersed by ascospores that infect leaves and rachises, resulting in lesions that can spread into twigs, stems and branches causing stem lesions and cankers, often resulting ultimately in the weakening and death of affected trees (Gross, Holdenrieder, Pautasso, Queloz, & Sieber, 2014).

Most studies of pathogenicity of *H. fraxineus* have followed the method of Kowalski and Holdenrieder (2009). The fungus is grown on malt agar for 3 weeks, then small ash wood sticks are placed on the fungal colony and incubated for another 3 weeks. These sticks are then inserted into an incision in the bark with one inoculation point per tree. Commonly, lesion development is assessed by destructive sampling several months after inoculation. A similar method has been applied to rachis inoculations on intact ash leaves (Kowalski, Holdenrieder, & Bilański, 2015; Schwanda & Kirisits, 2016). More recently, Mansfield, Galambos, and Saville (2018) were successful in inducing infection of leaves following inoculation with quantified ascospores numbers in a water suspension. As well as lesion length, Schwanda and Kirisits (2016) recorded leaf wilt and shedding of leaves after rachis inoculations over a period of 2–4 months. Inoculations without wounding through leaf scars have also been used but with less success (Kräutler, Treitler, & Kiritis, 2015).

Here we describe a method of rachis inoculation, which allows several replicate inoculations on the same tree. We show that disease progression can be tracked over time and present a five‐point scale for recording symptoms on leaves. This method reduces the assay time from months to weeks.

## 2 MATERIALS AND METHODS

Nineteen *H. fraxineus* isolates were used in in vitro experiments, of which five were also used in pathology experiments (17001, 17016, 17031, 17048 and 17064). Isolates were collected from individual lesions on stems of *F. excelsior* in three ashwoods in Norfolk, England in 2017, and cultured on ash leaf malt agar (ALMA; Gross et al., 2012, amended to 30 g of ash leaf tissue per litre). To assess growth rate, the isolates were grown on both ALMA and malt extract agar (MEA; 2% malt extract) at 20°C in the dark with three replicates on each medium. Colony diameter on agar was measured at 7, 10 and 15 days after inoculation.

Ten ash seedlings used for inoculation experiments were obtained from separate locations in Norfolk, UK in 2013 and kept in 3 L pots in an unheated glasshouse. The plants were free of dieback symptoms (Gross et al., 2012) when collected and were monitored closely thereafter. They were pruned in 2016 to restrict their height to approximately 1.2–1.5 m. Stems had a diameter of 7–10 mm at 50 cm height from the soil surface at the time of inoculation. Approximately 1 cm^2^ of the fungus was excised from the edges of 7‐ to 10‐day‐old cultures on ALMA, placed in a 2‐ml Eppendorf tube and mashed thoroughly using a scalpel blade. To inoculate the ash seedlings a 10 mm long slice was cut into the epidermal layer of a rachis between the 3rd and 4th pairs of leaflets, with the rachis still remaining attached at one end to the plant. Approximately 10 mg of the mycelium agar mix was then placed under the flap and Parafilm^®^ M wrapped around the wounded area. Each of the five seedlings in each experimental replicate was inoculated with three of the five isolates, with each isolate replicated three times. Each seedling also had three control mock inoculations of agar only, making a total of 12 inoculated leaves per tree. The first test was carried out in May 2017, and the whole experiment was repeated on a different set of five seedlings in July 2017. Each isolate was applied to at least two trees in each experiment. Details of the experimental design and data are available at www.datadryad.org.

Aggressiveness was assessed by measuring from the centre of the rachis wounding point to the end of the lesion both proximally (towards stem) and distally (towards leaf tip). The measurements were made on six occasions from 10 days after inoculation (dai) to 28 dai. Observations on the development of specific symptoms were also noted at each timepoint and converted to a numerical scale of Progress (see Section 3 and Table 1). This scale included recording whether a leaf had abscised and the appearance of the leaf. Where possible, re‐isolation of the fungus from the rachis was attempted.

**Table 1:** Statistical analysis of in vitro growth and *in arbore* disease progress and lesion length of *Hymenoscyphus fraxineus*, the ash dieback pathogen of *Fraxinus excelsior*

(a)			
Variable	*df*	m.s.	*p*
Medium	1	6.8750	<0.001
Isolate	18	4.0416	<0.001
Medium.Isolate	18	1.0204	0.04
Residual	72	0.5631	

(b)			
Variable	Growth on MEA	Lesion length	Disease progress
Growth rate on ALMA from days 10 to 15 (mm/day)	0.93	0.86	0.99
Growth rate on MEA from days 10 to 15 (mm/day)		0.88	0.95
Proximal lesion length on rachis (mm)			0.91

(c)					
Variable	*n*.*df*	Disease progress		Proximal lesion length	
		*F*	*p*	*F*	*p*
Experiment	1	24.84	<0.001	71.53	<0.001
Isolate	4	3.85	0.009	25.53	<0.001
Expt.Tree	8	1.23	0.3	46.64	<0.001
Expt.Isolate	4	2.64	0.05	22.46	<0.001
Expt.Tree.Isolate	12	1.31	0.2	22.26	0.06

(a) Analysis of variance of in vitro growth rates on agar media, ash leaf malt agar (ALMA) and malt extract agar (MEA). Data were the radial growth rates (mm/day) of 19 hr*. fraxineus* isolates between 10 and 15 days after inoculation of the plates. (*df*: degrees of freedom; m.s.: mean squares; *p*:* F*‐test probability.). (b) Correlation coefficients between mean in vitro growth rates and pathogenicity variables for five isolates of *H. fraxineus*. (c) Fixed effects in linear mixed models of disease progress (0–4) and proximal lesion length (mm) between 10 and 28 days after inoculation of *Fraxinus excelsior* trees with *Hymenoscyphus fraxineus*. Five trees were inoculated with the same five isolates in each of two experiments. Lesion length was analysed for leaves with progress values of 1, 2 or 3. For each variable, an autoregressive order 1 repeated measures model was fitted with the individual leaf as the residual stratum. Length data were log_10_ transformed for the purpose of statistical analysis. Denominator *df* = 44.1 for progress and 51.6 for length. (n.*df*: numerator degrees of freedom; *F*: Wald statistic ÷n.*df*;* p*:* F*‐test probability.)

Data on in vitro growth rates were analysed by general linear modelling. Data were isolates’ radial growth rates (mm/day) from 10 to 15 days after inoculation of the ALMA plate; after 15 days, some isolates reached the edge of the Petri dish. Data on disease progress and growth of the lesions were analysed using a linear mixed model of repeated measurements fitted by residual maximum likelihood. The first‐order autoregressive model was used because it had the lowest deviance of the models available. Fungal growth data were transformed to log_10_(lesion_down+1), where lesion_down was the distance (mm) from the wounding point to the promixal edge of the lesion. Statistical analysis (Table 1) was done with the package GenStat 18th Edition (VSN International, Hemel Hempstead).

## 3 RESULTS AND DISCUSSION

**Figure 1:**
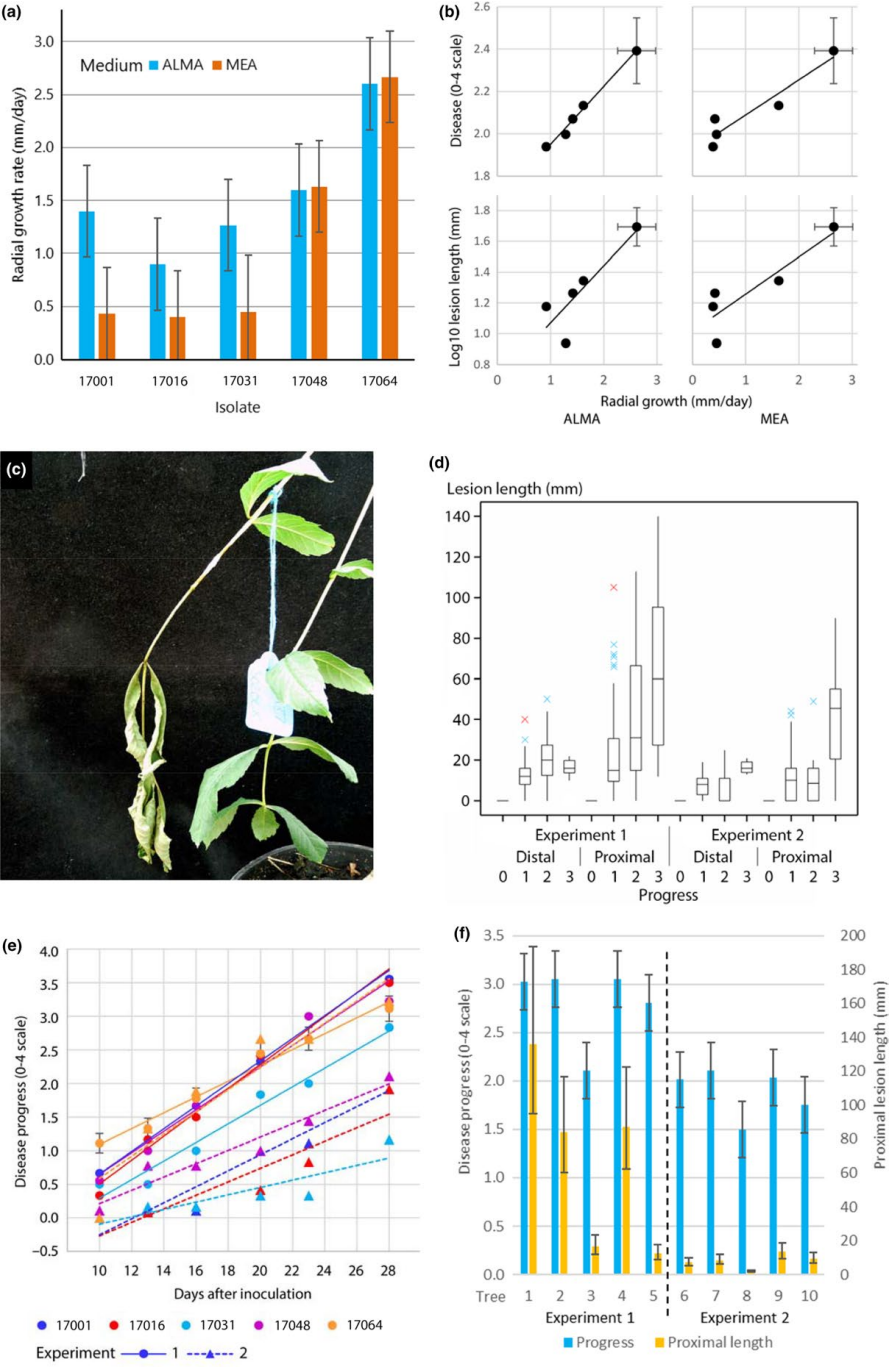
(a) Radial growth rate (mm per day) on ash leaf malt agar (ALMA) and malt extract agar of the five *Hymenoscyphus fraxineus* isolates used in inoculation experiments. Error bars are ±1 standard error (*SE*). (b) Relationship of disease progress (0–4 scale) and proximal lesion length (mm, log_10_ transformed) to in vitro growth rates of *H. fraxineus*. Each point represents one isolate. Representative error bars (±1 *SE*) are shown for isolate 17064. (c) An inoculated leaf at the top dead stage (progress score 3). The inoculation site, wrapped in Parafilm® M, is to the left of the tag. (d) Boxplot of distal and proximal lengths (mm) of lesions with Progress scores 0, 1, 2 or 3 at 28 days after inoculation in Experiments 1 and 2. Data are aggregated across isolates and trees. (e) Predicted mean disease progress (0–4 scale) of five *H. fraxineus* isolates in two experiments in each of which five trees were inoculated. (f) Predicted mean disease progress (0–4) and proximal length (mm) of lesions on ash seedling rachises infected with *H. fraxineus* at 28 days after inoculation. Trees 1–5 were tested in Experiment 1 and trees 6–10 in Experiment 2

The in vitro radial growth rate of 19 isolates, including the five used subsequently in inoculation experiments, was generally faster on ALMA (mean 1.59 ± 0.10 mm/day between days 10 and 15) than on MEA (1.11 ± 0.10 mm/day). The isolates varied considerably in mean growth rates and there was some variation in their relative growth rates on the two media (Table 1a, Figure 1a). Among the five isolates used in inoculation experiments, chosen to reflect the range of in vitro variation, growth rates on the two media were strongly correlated (Table 1b, Figure 1b).

In all inoculations, either a visible lesion developed or there were other symptoms such as leaf abscission or wilting; most inoculations produced both symptoms. None of the control, mock‐inoculated leaves developed lesions or foliar symptoms. Observations on symptom development were converted into a Progress variable:

0: *No lesion visible*;
1: *Visible lesion* with brown necrotic area on rachis;
2: “*Pre‐top dead*,” with some curling of the distal leaflets and a slight bend appearing in the rachis;
3: *Top dead*, with wilting and drying of the distal leaflets, often accompanied by a distinct bend of the rachis; the lesion may continue to develop proximally even if the distal part has died (Figure 1c);
4: *Leaf abscission*, with all or part of the leaf abscising from the plant.

This scale was used to analyse the progress of each lesion over the course of the experiment. Leaf abscission occurred frequently in Experiment 1, in 22 of 44 inoculated leaves, but less often in Experiment 2, in only 4 of 45 leaves. In all but one inoculation, a necrotic lesion developed but in one case, a leaf dropped without associated lesion development (Experiment 1, Tree 3, 16 days after inoculation with isolate 17048); this effect was scored as Progress = 4. A lesion developed on most leaves before the pre‐top dead symptom was noted, but one leaf in Experiment 1 and two leaves in Experiment 2 developed this symptom before lesion formation and were scored as Progress = 2; each of these leaves was inoculated with a different isolate. There was significantly more extensive symptom development in Experiment 1 than Experiment 2, with greater disease progress and longer lesions (Table 1c, Figure 1d–f).

Lesions were generally longer on the proximal side of the inoculation site, nearer the stem, than on the distal side nearer the leaf tip (Figure 1d). There was significant variation between isolates in lesion length (Table 1c) and the mean proximal lesion length was closely correlated with isolates’ growth rates on both ALMA and MEA (Table 1b, Figure 1b). All isolates except 17064 were less aggressive in Experiment 2 (Figure 1e). There was highly significant variation in mean lesion length between seedlings (Figure 1f). Although there was a great difference in proximal lesion length between the two experiments, this was less striking for disease progress (Figure 1f). There was no significant evidence for variation in the susceptibility of individual trees to different *H. fraxineus* isolates (Expt.Tree.Isolate term in Table 1c). Both measures of symptom development were highly correlated with in vitro growth rate (Table 1b, Figure 1b).

Re‐isolation of *H. fraxineus* was attempted from 75% of samples in Experiment 1% and 40% in Experiment 2. In the remainder of the leaves, either slow disease progress meant it was not possible to sample an isolate away from the original inoculation point, or there were too many contaminating organisms. There was a positive identification of *H. fraxineus* in 94% and 83% of samples in the two experiments respectively, based on colony morphology. In the remaining samples, while the lesions had the appearance of an *H. fraxineus* infection, re‐isolation of the pathogen was unsuccessful, probably because it was inhibited by competing microorganisms.

The inoculation method reported here, using mycelium grown on agar media, can be used reliably and repeatedly to compare the pathogenicity of different *H. fraxineus* isolates to *F. excelsior* and may also be applicable to other *Fraxinus* species. It could also be used to compare the susceptibility of different accessions of *F. excelsior* to dieback and thus evaluate the role that restriction of symptom development in the rachis may play in the expression of dieback resistance.

The method avoids the use of ash wood sticks, which requires availability of a suitable wood source and time to make, autoclave and inoculate the sticks. The overall preparation time for the assay was therefore reduced from about 6 weeks to about 7 days. The present assay on seedlings took 28 days, which is considerably faster than previously reported experiments on this pathosystem (Kowalski et al., 2015; Schwanda & Kirisits, 2016). This method also enables multiple isolates and inoculation points to be placed on the same ash seedling, thus reducing the number of seedlings required depending on the purpose of the experiment. It also allows statistically robust experiments because the effect of an isolate on a particular host genotype can be replicated without the need for clonal material. The generally slower disease progress in Experiment 2 suggests that the aggressiveness of four of the five *H. fraxineus* isolates may have become attenuated in in vitro, emphasizing the need to use fresh isolates when screening ash material for resistance to dieback. Alternatively, older leaves may have greater resistance to infection or disease aetiology may be affected by environmental conditions.

*Hymenoscyphus fraxineus* typically infects *Fraxinus* through the leaves so a versatile method of foliar inoculation enables the responses of the plant to infection of stems (Kowalski & Holdenrieder, 2009) and foliage to be distinguished. All five isolates used here induced lesion development in the rachises and there was no significant evidence for specific interactions between *H. fraxineus* isolates and *F. excelsior* accessions within experimental replicates. The absence of specificity at the level of host and parasite genotypes contrasts with the specificity of interactions between *Hymenoscyphus* and *Fraxinus* species (Kowalski et al., 2015).

This study adds to previous observations on development of symptoms following ash inoculations reported by Schwanda and Kirisits (2016) and Mansfield et al. (2018) by noting the “pre‐top dead” symptom, which is clearly the precursor to wilting of the distal end of the leaf. In two inoculations, this phenotype occurred when no rachis lesion was visible. In one inoculation the “leaf drop” symptom also occurred in the absence of a lesion. These observations imply that a rachis lesion is not the only indicator of disease, and that wilting and abscission are also potential symptoms. Also, sometimes the distal end of the leaf can senesce (“top‐dead”) but the lesion continues to develop proximally along the rachis. Recognition of diverse symptoms may be important if trees are observed at only a single timepoint.

Significant variation in disease progress between different ash seedlings occurred in both experiments, so the method described here can be used to rapidly assess potential variation in foliar resistance to ash dieback. The limited evidence for specificity of interaction of host and parasite genotypes suggests that it may be possible to use a small number of isolates of *H. fraxineus* to assess ash genotypes for foliar resistance. We recommend that isolates to be used for foliar resistance testing in the greenhouse or field should first be screened for vigorous in vitro growth; MEA is satisfactory for this purpose because while the growth of *H. fraxineus* isolates on MEA is slower than that on ALMA, the two were highly correlated. They should also be tested for strong proximal lesion development on a known susceptible tree, although it is expected that lesion growth will be highly correlated with growth on MEA. A few of the more aggressive isolates can then be selected for use in resistance tests; using at least three isolates would insure against an isolate having unexpectedly poor performance.
